# The Index-Based Subgraph Matching Algorithm with General Symmetries (ISMAGS): Exploiting Symmetry for Faster Subgraph Enumeration

**DOI:** 10.1371/journal.pone.0097896

**Published:** 2014-05-30

**Authors:** Maarten Houbraken, Sofie Demeyer, Tom Michoel, Pieter Audenaert, Didier Colle, Mario Pickavet

**Affiliations:** 1 Department of Information Technology, Ghent University - iMinds, Ghent, Belgium; 2 Division of Genetics and Genomics, The Roslin Institute - University of Edinburgh, Midlothian, Scotland, United Kingdom; Semmelweis University, Hungary

## Abstract

Subgraph matching algorithms are used to find and enumerate specific interconnection structures in networks. By enumerating these specific structures/subgraphs, the fundamental properties of the network can be derived. More specifically in biological networks, subgraph matching algorithms are used to discover network motifs, specific patterns occurring more often than expected by chance. Finding these network motifs yields information on the underlying biological relations modelled by the network. In this work, we present the Index-based Subgraph Matching Algorithm with General Symmetries (ISMAGS), an improved version of the Index-based Subgraph Matching Algorithm (ISMA). ISMA quickly finds all instances of a predefined motif in a network by intelligently exploring the search space and taking into account easily identifiable symmetric structures. However, more complex symmetries (possibly involving switching multiple nodes) are not taken into account, resulting in superfluous output. ISMAGS overcomes this problem by using a customised symmetry analysis phase to detect all symmetric structures in the network motif subgraphs. These structures are then converted to symmetry-breaking constraints used to prune the search space and speed up calculations. The performance of the algorithm was tested on several types of networks (biological, social and computer networks) for various subgraphs with a varying degree of symmetry. For subgraphs with complex (multi-node) symmetric structures, high speed-up factors are obtained as the search space is pruned by the symmetry-breaking constraints. For subgraphs with no or simple symmetric structures, ISMAGS still reduces computation times by optimising set operations. Moreover, the calculated list of subgraph instances is minimal as it contains no instances that differ by only a subgraph symmetry. An implementation of the algorithm is freely available at https://github.com/mhoubraken/ISMAGS.

## Introduction

In modern society, technology has been applied to create and study numerous advanced systems in various fields as biology, sociology, informatics and others. To understand their internal dynamics, many of these systems can be modelled using graph theory. By interpreting the systems as graphs of interconnected components, a vast array of network processing methods enables detailed analysis of the underlying, fundamental properties.

More specifically in biology, graphs are very well suited to model interactions between different proteins. A graph can be constructed by modelling proteins and interactions among them as nodes and edges respectively. A powerful analysis technique is described in [1] and consists of finding *network motifs* in the graph. These network motifs denote small interactions patterns between several proteins that are unusually more present in the graph than expected by chance. They can be modelled as small subgraphs which can then be searched in the larger network representing all known interactions between all proteins. By discovering these network motifs, our understanding of the underlying mechanisms of the network can be improved.

To find these network motifs, several tools and algorithms have been developed. Mfinder [2] was one of the early tools to mine graph data for network motifs. Similarly, the FANMOD [3] tool was developed which, compared to Mfinder, improves performance by using the RAND-ESU algorithm [4]. It uses unbiased sampling of subgraphs to speed up the calculations and includes isomorphism tests by using the Nauty [5] isomorphism tools which offer a description of the internal symmetry of the subgraphs. More advanced network motif finding techniques, focusing on graph properties and data structures, are proposed in [6] and [7]. G-Tries [6] are multi-way trees that encode the set of subgraphs/network motifs to be found in a single data structure. When two subgraphs that have to be enumerated have a common substructure, the matching for the substructure can be done simultaneously, speeding up queries significantly compared to doing both searches separately. In contrast to speeding up the network analysis by combining all different tests, Grochow and Kellis [7] optimise the individual subgraph matches by generating symmetry-breaking rules to prune the search space. By incorporating the symmetry of the subgraph in their search, they reduce the search space exploration and speed up queries.

The algorithms mentioned above use subgraph enumeration to find network motifs in the network. However, a different but related network analysis approach [8] is based on calculating graphlet degree distributions. A *graphlet* is a small connected non-isomorphic induced subgraph in a larger network for which the instances will be counted. However, contrary to network motifs which are partial subgraphs, graphlets are induced graphs, which means that if an edge is absent in the graphlet specification, it should also be absent in the graphlet instances and thus in the larger network. While graphlets and motifs are defined differently, they are both used to analyse networks by enumerating the graphlet/motif instances in the graph. The network motif analysis consists of finding unusually frequent subgraphs, whereas the graphlet-based analysis aims at characterising entire graphs by counting the occurrences for each graphlet from a predefined set. Similar to a node degree distribution, the counts form a distribution that represents the structure of the network in terms of graphlets. However, contrary to network motif analysis, the graphlets do not need to be over-represented (compared to random networks) [1]. As with network motif analysis, the graphlet analysis heavily relies on the enumeration of the graphlet instances which should be optimised.

While the discussed algorithms so far were developed to analyse full networks (by using network motifs and graphlets), the aim of this paper is to present a general subgraph matching algorithm. This algorithm can be used on its own to count or enumerate all specific occurrences of a subgraph in a larger network but can also be used as a building block for a full network analysis algorithm. Such an algorithm needs to be carefully designed as the subgraph isomorphism problem is proven to be NP-complete [9], [10]. As network modelling is used in various applications, the subgraph isomorphism problem has many variants and several classes of algorithms exist for solving it. In this paper, we focus on exact algorithms for which a strict correspondence between the specified subgraph and the requested instances in the graph is required. Well-known algorithms in this class are the Ullmann [11], the VF [12] and the VF2 [13] algorithms. Ullmann uses a matrix-based representation of the search space and iteratively prunes uninteresting branches in the search tree. Pruning is done by applying a refinement procedure to eliminate candidate nodes (for mapping to a subgraph node) based on the neighbours of the candidate and the required connectedness to the neighbours of the subgraph node. While the Ullmann algorithm is versatile, as it can be used in a wide range of isomorphism problems, it is matrix-based, which causes high memory requirements. Less memory is required by VF and VF2 algorithms which are graph-based. These algorithms search the network by creating an initial partial mapping between the source graph ( = the large network) and the subgraph ( = the network motif) and iteratively generating candidate pairs to be added to the mapping. Aside from speeding up the search, the graph modelling in VF2 significantly reduces the memory requirements as it only requires 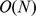 memory while Ullmann requires 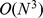. As biological networks tend to be very large (millions of nodes in some applications), reducing the memory requirements allows for a greater applicability.

In previous work, the Index-based Subgraph Matching Algorithm (ISMA) [14] was presented and compared against the above-mentioned subgraph matching algorithms. Like VF2, ISMA also searches the source graph for subgraph instances by creating a partial subgraph-to-graph-node mapping and expanding it iteratively. However, ISMA intelligently determines the order in which the partial mapping is expanded and avoids unnecessary computations. These optimisations greatly reduce the search space and speed up query times. In this paper, we introduce the Index-based Subgraph Matching Algorithm with General Symmetries (ISMAGS) in which search space size and query times are further reduced by incorporating the internal symmetry of subgraphs as constraints into the algorithm. Our constraint-based pruning is similar to that in [7] in which the breaking of the symmetry is done by iterating the symmetry analysis during the search. Based on the partial mapping constructed at that point, constraints are generated to avoid exploring symmetric parts of the search tree. However, the symmetry analysis in [7] is repeated several times and requires generating an exhaustive list of isomorphisms of the subgraph. In ISMAGS, only one symmetry analysis is needed to obtain a compact set of generating permutations and constraints to break the symmetry. Compared to [7], we also present results for larger networks with multiple edge types.

In the rest of this paper, we first briefly outline the ISMA algorithm and its functionality for incorporating simple symmetric structures. As only basic symmetric relations are incorporated by ISMA, we then continue by presenting our approach to incorporating symmetry in the search tree. After explaining how ISMA deals with symmetry, the symmetry detection in ISMAGS is presented and validated in a group-theoretical context. Subsequently, we show the derivation of the symmetry-breaking constraints and integrate them into the global algorithm. We then show the performance gain of ISMAGS over ISMA for multiple networks with various properties (size, edge types) and various subgraphs. We also compare against the VF2 algorithm and (parts of) the G-Trie and the Grochow-Kellis algorithm (as the full algorithms were developed to find network motifs while ISMAGS is only concerned with the subgraph enumeration method).

## Methods

The following section contains a brief introduction to the core aspects of ISMA, followed by closer examination of its internal symmetry handling. Next, the core features of ISMAGS to take into account all symmetric structures are presented along with a description of the global algorithm.

### ISMA

In previous work [14], ISMA was developed to find matches for composite network motifs (subgraphs with type-annotated edges) in large graphs by dynamically optimising the order in which nodes are investigated during the search process. More formally, the algorithm searches in a graph 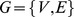 with 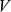 denoting the set of nodes and 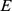 denoting the set of edges. Each edge 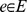 can be represented by a triplet 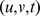 with 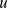 and 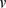 the start and end node respectively and the type 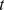 of the edge, defining properties such as whether it is directed or undirected.

Adopting the terminology of [14], a subgraph 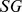 is defined as a string of tokens representing the specific subgraph topology. As (anti-)parallel edges are not allowed, a subgraph of 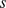 nodes has a maximum of 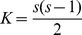 edges and can be represented by a string of 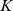 tokens, with each token encoding the type of the edge (including the direction) at that position. The first token in a subgraph string denotes the edge going from node 1 to node 2, the next 2 tokens denote the edges going from node 1 and 2 to node 3 and so on. The string of tokens “ABCDEF…” thus denotes a subgraph with an edge of type A from node 1 to node 2, an edge of type B from node 1 to node 3, an edge of type C from node 2 to node 3, an edge of type D from node 1 to node 4 and so on. A more elaborate description is given in [14].

In ISMA, the main goal is to reduce the search space by carefully selecting the next node to be matched. At any given stage in the search process, a partial mapping of graph nodes (in 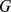) to subgraph nodes (in 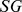) is maintained. Initially, the mapping is empty as no nodes have been matched. The algorithm then selects a subgraph node based on the number of candidate nodes in 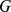 that can be mapped on that node. At the start of the algorithm, the candidate set for each node is constructed based on the edges arriving/departing in/from that node. For example, if the first node 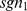 in the subgraph has an outgoing edge of type 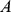 and an incoming edge of type 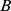, the candidate set 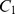 is calculated as the intersection of the set of nodes in 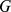 with outgoing edges of type 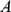 and the set of nodes in 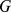 with incoming edges of type 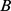. The subgraph node (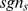) with smallest candidate set is then selected to investigate next.

Once 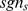 is found, the (partial) mapping is expanded by iteratively mapping every graph node 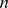 in its candidate set to 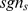. Mapping 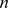 to 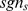 introduces new constraints for the rest of the subgraph instance: if a subgraph node 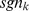 is connected to 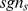 with an edge of type 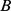, the graph node mapped to 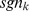 also has to be connected to 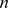 with an edge of type 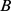. To select the next node to investigate, the constraints are incorporated in the candidate sets by intersecting the old candidate sets with the neighbour set of the newly mapped node 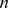. The mapping process can then repeat for each node in the subgraph. By iteratively mapping and backtracking, all subgraph instances in the graph are found.

The subgraph node selection method is optimised in ISMA to avoid unnecessary set operations. As described above, the candidate set 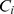 is calculated as the intersection of a number of other sets. Instead of calculating all 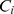 sets (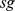 in total) each requiring intersecting a few sets to find the smallest candidate set, ISMA keeps track of the smallest set for each subgraph node. The size of this set is a heuristic estimate for the size of 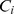. The algorithm is further optimised by using custom data structures for set operations.

### Symmetry in ISMA

While ISMA can find all subgraph instances in a graph for any subgraph, it was designed to exploit symmetric properties. While a brief description of the symmetry-handling in ISMA is given here, the reader is referred to [14] for the full approach. Two nodes have a *reflection symmetry* if and only if they can be switched without changing the subgraph topology. If a subgraph contains two reflection symmetric nodes 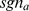 and 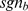, the graph nodes 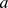 and 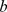, mapped to 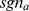 and 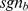 respectively, can be switched with the result being a valid subgraph instance. This property can be exploited and allows to only examine half of the search space.

The symmetry is exploited in ISMA by adding extra constraints to the candidate set generation. If subgraph node 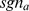 is present in the partial mapping, the algorithm takes this into account when mapping 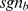. ISMA will prohibit nodes, that were previously mapped to 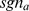, to be considered for mapping onto 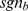.

When the candidate set 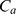 of subgraph node 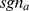 is determined, it will consist of all nodes that are valid to be mapped onto 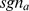 and by symmetry also onto 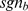. As described above, ISMA will iteratively add every node in 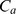 to the partial mapping and continue mapping the rest of the nodes. Every time a new node from 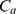 is examined, it is removed from 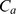 before the search is continued. When the candidates for 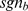 are determined, ISMA will use 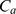 as a constraint to force nodes in 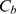 to be in 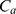. This will avoid generating the symmetric counterparts of the subgraph instances. If 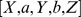 was previously examined, its counterpart 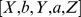 will not be examined as, at that time, 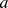 will no longer be in 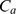 and therefore not in 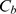.

**Figure 1:**
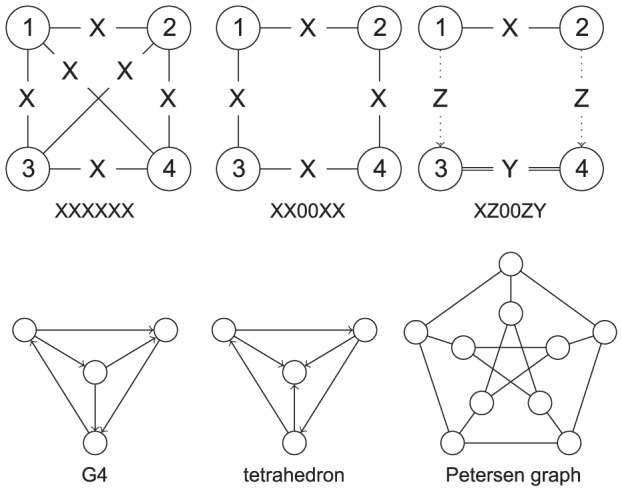
Subgraph examples. In “XXXXXX”, every node is symmetric to every other node as, in a clique, all nodes can be swapped. The “XX00XX” graph is symmetric as it has rotation symmetry (all nodes can be shifted in the ring) and reflection symmetry (top two nodes can be switched with bottom two nodes for example). The “XZ00ZY” graph is also symmetric as the same configuration is obtained when node 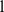 is switched with node 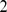 and node 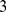 with node 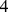. While the G4 graph has no symmetric properties, the tetrahedron and the Petersen graph [25] have more complex symmetric structures.

The same basic principle is also applied to cyclic rotations. A subgraph contains a *cyclic rotation symmetry* if and only if it has a sequence of nodes that can be shifted without changing the subgraph configuration. An example can be seen in the “XX00XX” subgraph in Fig. 1.

### Symmetry in ISMAGS

**Figure 2:**
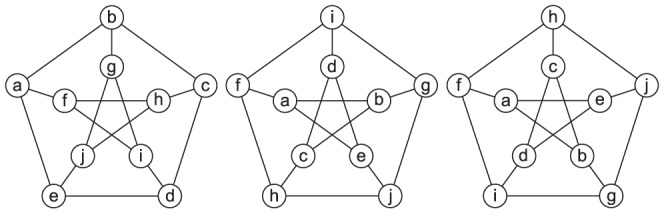
Some permuted instances of the Petersen graph.

While the symmetry handling approach in ISMA performs well for small subgraphs with small reflection or rotational symmetries, it cannot efficiently tackle larger subgraphs with more elaborate symmetric structures. ISMA was only optimized for simple symmetric structures that can be easily detected like reflection symmetries between 2 nodes ( = 2 nodes that can be switched) or ring structures ( = rotation symmetries). It eliminates similar subgraph instances induced by these symmetries but does not handle larger symmetric structures (consisting of multiple nodes being switched) or more complex symmetric structures (in which nodes can be part of multiple symmetric structures and the symmetric properties are less easily detected). As the symmetry analysis in ISMA was limited and did not extract complex multi-node symmetries, these were not taken into account which means that multiple similar subgraph instances induced by these symmetries will be returned. Fig. 2 shows valid subgraph instances that differ by only a permutation of the subgraph nodes. As the degree of symmetry increases, listing all possible permutations becomes very time-consuming. A more efficient approach would be to avoid finding the permuted instances by reducing the search space.

This section deals with the modified approach in ISMAGS to successfully handle all symmetric structures in a subgraph. The main idea of ISMAGS is to detect the symmetric properties in the subgraphs and convert them to pruning rules for the search space. Detecting the symmetries is detailed in the next paragraph, followed by the symmetry-breaking approach and a description of the data structures used. In general, *breaking symmetry* means that the information of the symmetries is used to develop rules or constraints to simplify the search and speed up calculations. By fully breaking the symmetry, the set of subgraph instances returned by ISMAGS is minimal as a single subgraph instance will only be exported once while similar subgraph instances induced by the subgraph symmetry are omitted.

### Symmetry detection

The first step in ISMAGS is to determine the symmetric properties of the subgraph under examination. While the subgraph isomorphism problem is NP-complete, several basic techniques have been developed to minimise the required work. These techniques and how they are used in ISMAGS to develop a custom symmetry-breaking approach are explained next.

#### Subgraph partitioning

The basis for the symmetry detection is derived from Nauty [5]. The basic mechanisms are explained here but the reader is referred to [5], [15] for more details. The analysis starts by grouping the subgraph nodes 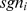 based on their incoming and outgoing edges as only nodes with similar properties could be symmetric to each other. The nodes are first grouped into an ordered partition 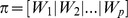 with every node in a cell 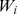 having the same number of outgoing/incoming edges to/from each of the other cells.

**Figure 3:**
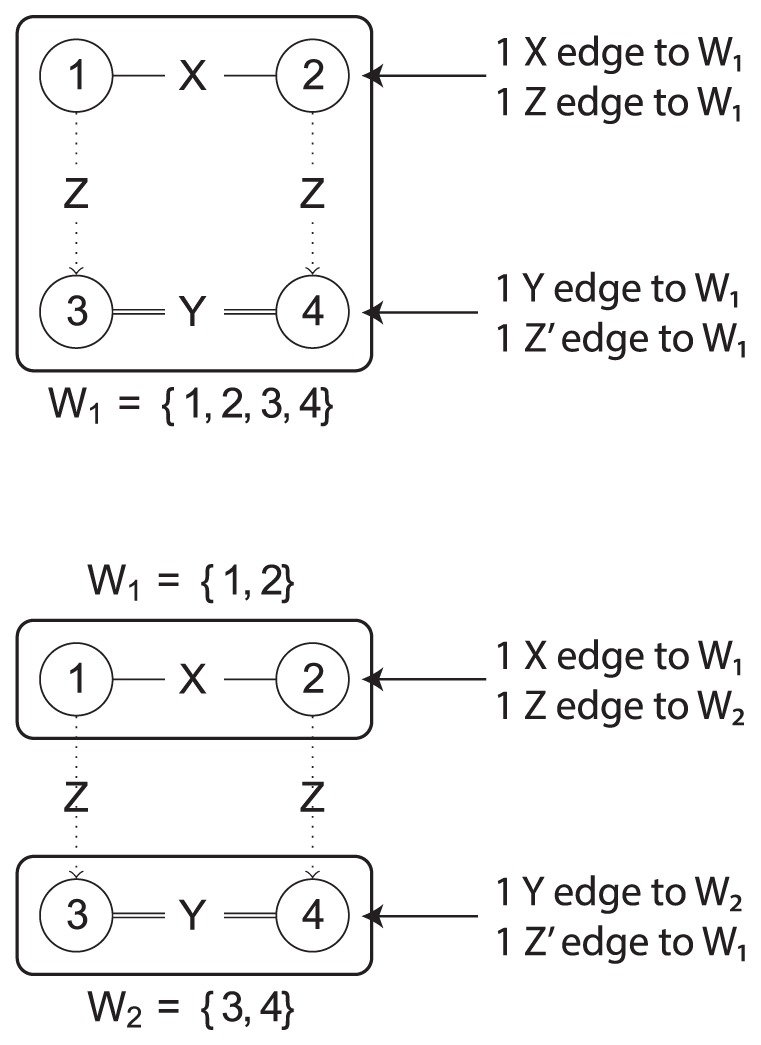
Example of subgraph refinement. The top figure shows the initial partitioning in the “XZ00ZY” graph in which all nodes are in the same cell. However, nodes 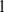 and 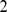 have an outgoing 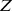 edge while nodes 3 and 4 do not. This indicates the partition needs to be refined. Nodes 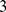 and 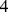 are put in a separate cell as shown in the bottom figure.

Fig. 3 illustrates how these partitions are formed. In the initial partition, all nodes are put in the same cell 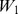. Every node is then analysed by determining the source/target cells of its edges. Nodes 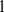 and 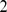 both have 1 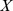-edge to node in 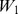 and 1 outgoing 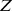-edge to another node in 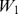. However, the top 2 nodes have different edge properties compared to the bottom 2 nodes. If nodes within one cell do not have the same properties, the partition needs to be *refined*. Cells containing nodes with different properties are split to ensure partition validity. The top nodes of the subgraph are separated from the bottom nodes by introducing a new cell. This leads to the partition in the bottom of Fig. 3. The nodes are then again analysed by their edges as the introduction of new cells can require splitting up other cells. This process is repeated until all nodes in the same cell have the same properties.

#### Ordered partition pair

**Figure 4:**
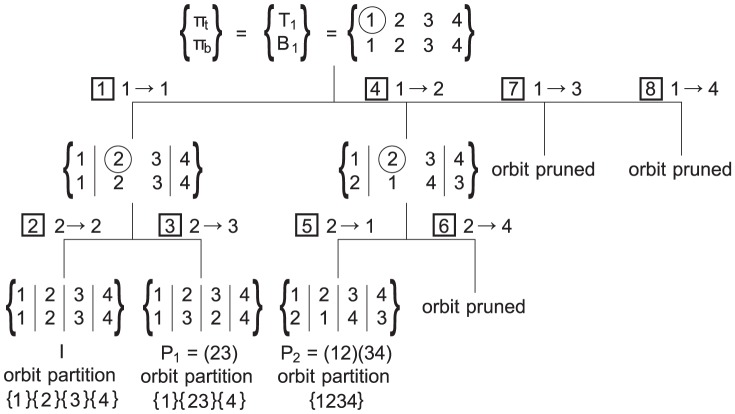
Subgraph symmetry analysis of “XX00XX”. The branches are denoted with the applied coupling. The boxed numbers indicate the order of tree traversal, with a depth-first exploration, according to the *smallest node first* coupling. The initial partition has all nodes in the same cells 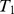 and 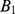. The first coupling 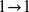 splits up the cells in both partitions in 3 cells (separation of cells denoted by |). When a permutation is found, the orbit partition is updated as shown. Orbit pruning is used to reduce the required computations as explained in text.

The actual subgraph symmetry analysis uses 2 partitions 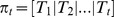 and 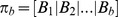 to analyse the subgraph. An example of the analysis can be found in Fig. 4. The 2 partitions together from an *ordered partition pair* (OPP). This OPP will be used to investigate symmetric structures by simultaneously refining both partitions. If a subgraph has symmetric structures, the refinement will have multiple branching points which lead to the symmetries in the subgraph. By exploring all branches during the analysis, all symmetries can be found.

#### Coupling

The 2 partitions in the initial OPP are identical and follow from the initial partitioning of the subgraph. Fig. 4 shows the symmetry-breaking algorithm for the “XX00XX” subgraph. The partitioning in the initial OPP at the top of the search tree consists of a single cell as all nodes have 2 edges to other nodes. The different branches in the OPP search tree are then separately investigated by selecting one of the subgraph nodes in one of the cells of 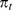 and mapping it to all subgraph nodes in the corresponding cell in 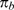. In the remainder of this work, each such mapping is referred to as a *coupling*. When coupling, the nodes are selected in the order of increasing node ID. The node with the smallest ID among all unmapped nodes gets selected first, both in the top partition (of which only 1 node is chosen) as in the bottom partition (for which coupling iterates over all nodes in order of increasing ID).

#### Recursive refinement

A coupling operation thus maps a node (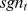) from a top partition cell to a node (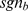) in the corresponding bottom partition cell. This is done in the partitions by putting 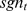 and 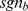 in a newly created cell in their respective partitions. The coupling is followed by a *refinement* operation on each of the partitions (top and bottom). As mentioned above, each partition groups nodes with identical properties. By introducing a new cell (for 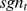 or 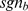), the grouping might no longer be accurate. Nodes with edges to/from 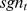 now have edges to/from the new cell while previously those edges were to/from the original cell of 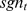 (analogously for the bottom partition with 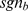). This change in properties needs to be incorporated in the partition, possibly yielding more cells to ensure that all nodes in one cell have the same properties. The partition is refined until it is completely valid again. Note that while the refinements of 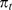 and 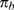 are done separately, each top partition cell will correspond to a bottom cell. The coupling and refinement operations are done recursively until all nodes have their own cell. At that point, there is a one-on-one correspondence between nodes in the top partition and nodes in the bottom partition, yielding a valid permutation.

In the example of Fig. 4, subgraph node 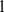 from 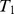 is coupled to the 4 possibilities in 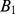. This splits up 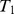 and 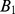 as node 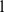 gets its own cell because of the coupling. It also leads to the further refinement of the initial OPP as in both partitions for nodes 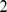 and 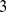, one of the incoming edges is coming from the new cell while for node 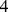 that is not the case. As shown in Fig. 4, the recursive refinement leads to the full subgraph analysis.

The key strength of using the OPPs in the analysis is that the discovered symmetry relations can be used to prune and speed up the analysis. When the coupling of a node in 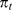 to a node in 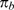 leads to 2 partitions with a different number of cells, the configuration can be discarded as no symmetries can be found. Even more extensive pruning is used in the original Nauty algorithm and its successors but is simplified here as the analysis in ISMAGS is combined with symmetry-breaking constraints (see next section) that modify the search process.

#### Orbit pruning

Orbit pruning [5] is a group theoretical optimisation technique to narrow down the search space. During the analysis of the subgraph, a set of generating permutations is built for the automorphism group 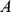 of the subgraph. In the example of Fig. 4, the set of generating permutations consists of 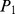 and 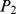. The automorphism group 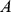 is a permutation group and will act on the set 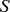 of all possible permutations on the subgraph nodes. The effect of 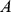 can now be analysed in 2 ways.

First, the effect of permutations 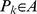 on individual subgraph nodes 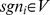 is considered. Starting from an element 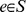, the permutation permutes the individual nodes to different positions. In general, the element 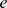 is transformed in 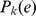 by permuting the individual subgraph nodes in the element.
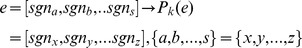
(1)The image 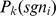 of 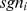 under 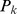 can then be used to define the *orbit partition*. This is a partition of 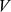 into disjoint cells 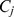 for which holds that
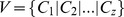
(2)

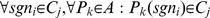
(3)More intuitively, when an automorphism is applied, a node can only be mapped to itself or another node in its orbit partition cell. Every time a permutation is found during the symmetry detection, the orbit partition is updated by merging the orbit partition cells of nodes that can be mapped to each other. In Fig. 4, 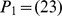 is found as an automorphism. The image 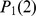 of node 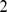 is node 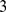 (and vice versa) and so they will share the same cell in the orbit partition.

For the second analysis of the effect of 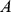, consider the relations between the elements of 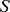. Given an element 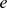 of 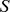, its *orbit* is the set of all elements of 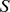 that can be found by combining all permutations in 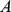 to 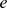. The orbits themselves form a partition of the set 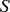 of all possible permutations on the subgraph nodes.
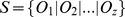
(4)

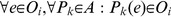
(5)


Orbit pruning relies on this partitioning to prune parts of the search space. As one element suffices for generating the entire orbit (by applying the generating permutations), the analysis can omit parts of the search space that would result in redundant generators. The orbit partition is used to detect these cases and to abort the search. A more detailed application of orbit pruning can be found in [16].

Without orbit pruning, the subgraph symmetry analysis described above continues finding all permutations after the necessary set of permutations is already exported. As only that set is needed to generate all permutations, orbit pruning is used to reduce the search space. In the example of Fig. 4, the orbit partition gets updated when permutation 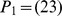 is found and again when 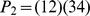 is exported. For 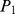, node 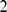 and node 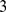 are put in the same cell in the orbit partition while 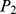 merges the cell of node 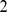 with the cell of node 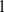 and the cell of node 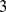 with the cell of node 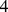. This results in all nodes being in a single cell. When the subgraph analysis is continued after finding 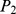, node 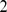 in the top partition would be coupled to node 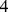 in the bottom partition but as node 2 and 4 share the same cell in the orbit partition, the coupling can be pruned. Backtracking further in the analysis would lead to top node 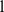 being recoupled to bottom nodes 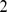, 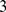 and 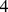 but this can be omitted analogously.

### Symmetry breaking

The symmetry detection in ISMAGS results in a set of permutations of the subgraph nodes and an orbit partition. The permutations can be applied on any valid subgraph instance to produce other valid instances. To avoid generating and exporting instances that can be obtained through permuting previously found instances, the symmetry needs to be broken. To explain the symmetry breaking in ISMAGS, some group theoretical concepts first need to be introduced.

#### Stabilisers

In general, permutations in a permutation group 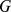 act on sequences of nodes by switching/swapping nodes to different positions in the sequence. However, some permutations do not change all nodes and leave some nodes on their original position. These permutations can be used to define *stabilisers*. For every node 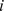, the stabiliser 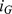 is defined as
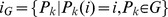
(6)For the example of the “XX00XX” subgraph with generating permutations 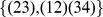, the stabiliser 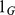 of node 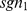 is 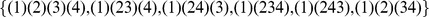 as these permutations all map node 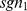 to itself.

#### Stabiliser chains

Using the concept of stabilisers, a *stabiliser chain* of (sub-)groups 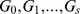 is defined for a permutation group 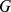, acting here on the set of subgraph node permutations. Each group in the chain is a subgroup of the previous (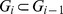) starting with 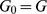. Formally, the groups are defined as
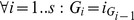
(7)More intuitively, the permutations in 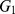 are those permutations that do not change node 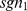 while the permutations in 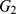 are those permutations in 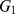 that do not change node 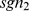 and thus leave 2 nodes unchanged.

#### Coset representatives

The *coset representative set* 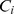 of a subgraph node 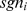 is defined as the set of subgraph nodes 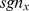 to which 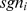 can be mapped in 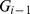.
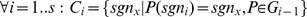
(8)Given 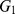 in subgraph “XX00XX”, 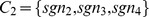 as 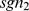 can be mapped to any subgraph node in 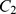 by the permutations in 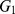.

Given the above definitions, the subgroup 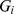 consists of those permutations that leave the first 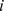 nodes unchanged. As shown in [17], stabiliser chains can be converted to symmetry-breaking constraints that fully break the symmetry of the subgraph. However, contrary to [17], the 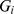 groups are not fully generated in ISMAGS. To generate the constraints, only the coset representatives for each subgraph node are needed. These coset representatives are generated during the symmetry analysis phase.

#### Determining coset representatives

Recall that during the symmetry analysis, nodes are coupled according to increasing ID. In the search tree, constructed by the couplings, the first leaf node to be reached is the identity permutation. When backtracking starts, the couplings are undone by replacing the last coupling, say 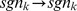, with a new coupling, say 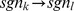. Undoing the mapping can be interpreted as looking for permutations that leave the first 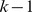 nodes in place but permute the remainder of the subgraph nodes. If such a permutation is found, it belongs to the 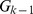 group. In Fig. 4, when 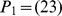 is found, it is a permutation leaving the first node unchanged and thus belongs to the 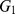 group. As detailed above, when a permutation is found, the orbit partition is updated. After 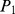 is found, the orbit partition cell 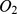 of node 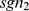 will be merged with the cell of node 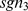.

Subsequently, the algorithm continues by backtracking and uncouples 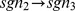. As all possible couplings for 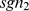 are tested, ISMAGS further backtracks and uncouples 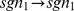. However, at this point in the analysis, the coset representative set 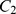 can be determined. Orbit partition cell 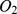 only contains subgraph nodes 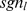 that can be mapped to 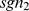 without remapping the first node as the first node has thus far remained mapped to itself due to the coupling. Cell 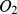 thus corresponds to the set of coset representatives of 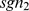.

Following the example above, the coset representative set 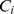 is found for every node as an intermediate orbit partition cell. Coset representative set 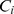 is set equal to its orbit partition cell when all possible mappings 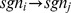 are evaluated and 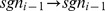 is to be undone next. While the orbit partition is updated every time a permutation is found, the 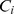 sets are not.

Note that not all nodes will have coset representatives generated. During the creation of the initial chain of couplings that results in the identity permutation, some nodes are mapped to themselves by the couplings while other nodes are mapped to themselves by the refinement procedure. For the latter nodes, no coset representatives will be generated as, with the first nodes fixed, they can only be mapped to themselves.

For the example in Fig. 4, 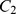 is set as 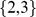 after 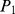 is found, right before 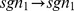 is undone. Set 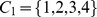 is found similarly at the end of the subgraph symmetry analysis, after 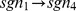 is investigated. Sets 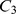 and 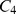 can be omitted as they can only be mapped to themselves if 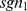 and 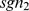 need to remain fixed.

#### Generating symmetry-breaking constraints

To break the symmetry in the subgraph, the coset representatives are converted into constraints on the IDs of the graph nodes mapped to the subgraph nodes, as shown in [17]. For every subgraph node 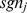 in the coset representative set 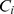 of subgraph node 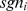, a constraint is introduced to force the ID of the graph node mapped to 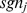 to be higher than the ID of the graph node mapped to 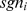. More formally, the following constraints are introduced to be used during the node mapping in ISMAGS.
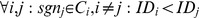
(9)


While the constraint generation is done as in [17], ISMAGS does not require explicitly generating the stabiliser chains (introduced by [18]) as only the coset representatives are necessary and found during subgraph symmetry analysis.

The approach to breaking symmetry in ISMAGS begins with a subgraph symmetry analysis derived from Nauty [16]. In general, Nauty generates a set of generating permutations for the automorphism group of the subgraph. However, the set of generating permutations generated is not necessarily unique. For the “XXXXXX” subgraph in Fig. 1, a generating set of permutations could be 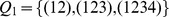 while 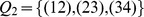, generated by ISMAGS, is equally as valid for generating the full set of permutations. By tuning the order of the node coupling, ISMAGS embeds the stabiliser chains into the search process. The permutations found are generators for as many subgroups 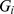 as possible while the coset representatives can readily be found. This eliminates the need for explicitly generating all possible permutations, the stabiliser chains and coset representatives as in [17].

### Integrating symmetry detection and symmetry breaking with ISMA

With the symmetry-breaking constraints described in the equation above, a full description of ISMAGS can now be given. While ISMAGS reuses the basic principles of ISMA to limit the search space, it goes much further in the subgraph analysis and pruning. A pseudocode description of the different steps is given in Table 1 in Algorithm 1. The actual subgraph instances are found and exported in the mapNodes function.

**Table 1:** Algorithm 1: findSubgraphInstances(Graph 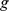, Subgraph 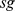).

1: Set<Constraint> 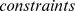 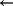 analyseSubgraph( 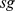 );
2: NodeListHandler[] 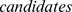 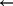 candidate node lists (to be intersected) for each subgraph node;
3: **for** SubgraphNode 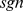 in 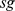 **do**
4: **for** Edge 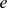 leaving/arriving in 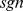 **do**
5: 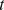 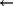 type of edge;
6: 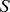 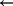 list of graph nodes in 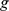 that have an edge of type 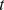 ;
7: add 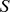 to 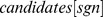 ;
8: **end for**
9: **end for**
10: SubgraphNode 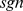 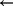 subgraph node with the smallest candidate node sublist;
11: NodeList 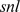 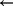 calculate candidate node list of 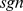 ;
12: mapNodes( 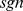 , 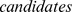 , 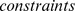 );

The search for all instances starts with the analysis of the subgraph specification for symmetric properties. This analysis is detailed in the previous sections and results in a set of constraints between the IDs of the graph nodes in a mapping. These constraints are stored for each subgraph node and used for determining the candidates for a specific subgraph node. After subgraph analysis, the search for subgraph instances, similar to ISMA, begins on line 3 of Algorithm 1 in Table 1 by creating the first candidate node lists based on the subgraph configuration. As in ISMA, the candidates for a subgraph node are determined by intersecting collections of nodes. However, the collections in ISMAGS are lists of ordered nodes based on node ID. Sorting of the lists of nodes and neighbours only needs to be done once for every network as the networks can be stored with the lists sorted. Using ordered lists accommodates quick subset selection (detailed further below) during node mapping. The candidates for a subgraph node are determined based on the edges in the subgraph specification. For each of its edges, the corresponding list of graph nodes is added to a set of lists. Once all lists are known, their intersection gives all graph nodes that have edges of the required types. Note that, as in ISMA, the intersection of the starting sets is delayed until after the subgraph node is selected to avoid calculating intersections that are not used.

Once the initial subgraph node is determined, the candidate node list is generated (see line 11 of Algorithm 1 in Table 1). This candidate node list is calculated with a linear sweep over the different (ordered) lists while all other intersections in ISMAGS are calculated by checking node membership to all lists (as in ISMA). As node lists are large at this point, a linear sweep is more efficient than node-by-node set membership tests.

The mapNodes function is very similar to the approach in ISMA and recursively maps graph nodes to subgraph nodes to find all subgraph instances. Every time a graph node is mapped to a subgraph node, the neighbours of the graph nodes are taken into account as new constraints for the remaining unmapped nodes. If graph node 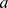 is mapped to subgraph node 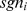 and 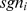 has an edge of type 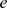 to subgraph node 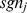, the candidate graph node for 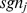 needs to have an edge of type 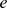 from 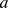. Note that the direction of the edges is taken into account in the edge type. Once the lists of neighbouring nodes are added to the constraint sets, the next node to investigate is determined.

The next subgraph node to examine is determined on line 13 heuristically as in ISMA. The intersection of lists is delayed to avoid unnecessary work. Instead, the lists that need to be intersected are stored separately per subgraph node. The size of the intersection is estimated by the size of the smallest list of that subgraph node. This is an upper bound on the actual size and trades off a slightly larger search space for less list intersections. To further optimise the calculation of the candidate list, ISMAGS disregards the lists introduced during initialisation (see Algorithm 2 in Table 2). These lists are generally very large (they list all nodes with an edge of the correct type) and contain little useful information, as the lists only indicates the presence of an edge which is verified each time a node is mapped. Omitting the lists results in shorter computation times during intersection with only a limited increase in search space size.

**Table 2:** Algorithm 2: mapNodes(SubgraphNode 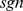, NodeListHandler[] 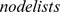, Set<Constraint>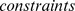).

1: List<Node> 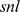 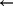 getCandidates( 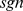 , 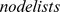 );
2: **for** Node 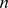 in 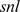 **do**
3: map 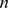 to 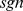 ;
4: **if** subgraph instance is complete **then**
5: export instance;
6: **else**
7: **for** Edge 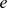 arriving/leaving 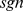 **to**
8: 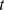 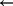 type of 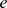 ;
9: 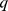 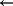 origin/destination of 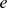 ;
10: 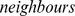 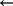 neighbours of 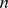 by type 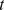 ;
11: addNeighbourList( 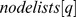 , 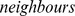 );
12: **end for**
13: 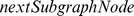 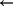 determineNextSubgraphNodeToProcess( 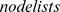 , 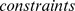 );
14: mapNodes( 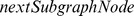 , 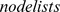 , 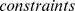 );
15: **end if**
16: unmap 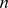 to 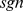 ;
17: **end for**

The key difference with ISMA is that when a subgraph node is selected for examination, the actual intersection is calculated using the symmetry-breaking constraints. The constraints are combined with the partial instance constructed so far to determine boundaries for the node ID of the graph nodes to be mapped on the subgraph node. If for the selected node 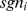 a constraint 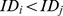 was generated and node 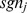 is already mapped, this gives an upper bound on the ID of a candidate for 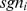. A lower limit is found analogously by considering the set of subgraph nodes which should have a smaller ID. The boundaries can then be used during intersection, allowing to skip the nodes with IDs outside the allowed range. As the lists to be intersected are sorted, binary search can be used to quickly find the start and ending point of the sublist of valid IDs.

While the symmetry breaking is the main source of the performance gain, additional speed-up could be gained by maintaining extra state. As explained above, the candidate sets are stored in memory as ordered lists to allow quick retrieval of nodes within a specific ID range. When lists are intersected, the intersection is determined by iterating over all nodes (in that valid ID range) and checking for membership of the other lists. In ISMAGS, this checking is done by using binary search on the sorted lists. To speed up this operation, a copy of the list could be maintained in a hash-based set. This would allow to check membership in overall constant time (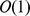), whereas binary search requires logarithmic time (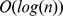, 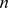 = number of entries in the list). Experiments show that an additional 5% speed-up could be gained by using this optimisation at the cost of almost doubling memory requirements. As memory is often a bottleneck in biological networks, the optimisation was not included in the presented version of ISMAGS.

The basic pseudocode of the subgraph analysis (to generate the constraints) is given by Algorithm 3 in Table 3. The initial OPP is constructed based on an initial partitioning of the input subgraph as detailed in the symmetry detection section above. The OPP is then recursively refined to find all symmetries (and constraints), as described in the two previous sections. As mentioned above, ISMAGS tunes the order in which the nodes are coupled in the OPPs by always selecting the node with the lowest ID first as shown in lines 5 and 9 of Algorithm 4 in Table 4. The constraints are generated on line 17.

**Table 3:** Algorithm 3: analyseSubgraph(Subgraph 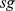).

1:Set<Permutation> 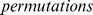 ;//create new set to store permutations
2: Set<Constraint> 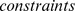 ;//create new set to store constraints
3: Set<Set<SubgraphNode>> 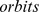 ;//create the initial orbit partition
4: **for** SubgraphNode 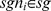 **do**
5: add set 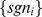 to orbits;
6: **end for**
7: Partition 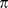 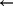 create initial partition of subgraph 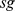 ;
8: OPP 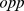 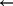 create initial OPP from 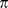 ;
9: processOPP( 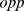 , 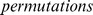 , 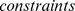 , 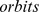 );
10: **return** 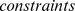 ;

**Table 4:** Algorithm 4: processOPP(OPP 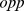, Set <Permutation>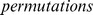, Set<Constraint>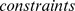, Set<Set<SubgraphNode>>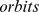).

1: **if** all nodes are mapped **then**
2: add current mapping to 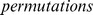 ;
3: update 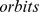 ;
4: **else**
5: 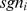 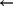 subgraph node with the lowest ID among the unmapped nodes;
6: 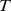 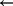 cell in top partition which contains 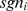 ;
7: 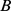 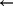 cell in bottom partition corresponding to 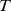 ;
8: sort 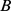 by increasing ID: [ 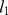 , 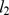 ,…, 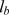 ];
9: **for** 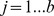 **do**
10: couple 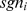 to 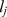 ;
11: 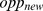 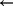 refine 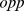 ;
12: processOPP( 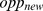 , 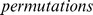 , 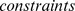 , 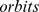 );
13: **end for**
14: **if** 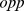 maps 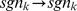 , 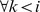 **then**
15: 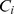 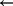 orbit of 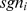 ;
16: **for** 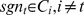 **do**
17: add 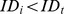 to 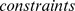 ;
18: **end for**
19: **end if**
20: **end if**

## Results

To illustrate the performance of ISMAGS, the algorithm is benchmarked against previously published results and algorithms. After a description of the algorithms and network data use, ISMAGS is compared against it predecessor ISMA to show the effects of the added symmetry breaking and related optimisations. In addition, ISMAGS is compared against the VF2 algorithm and the subgraph enumeration algorithms of Grochow-Kellis [7] (denoted by GK) and the G-Trie algorithm [6].

### Algorithms

The ISMA and ISMAGS algorithms were implemented in Java (version 1.6.0_26) while for the VF2 experiments, the VFLibrary (http://mivia.unisa.it/datasets/graph-database/vflib/) was used. To perform the GK experiments, the authors of [7] provided the original (Java) code for their algorithm from which the code for subgraph enumeration was extracted. This was necessary as the GK algorithm is a network motif finding algorithm while we present a subgraph enumeration algorithm. The experiments for the G-Trie results were done using the reference implementation on the homepage of the G-Trie author (http://www.dcc.fc.up.pt/gtries/). An implementation of the ISMAGS algorithm is freely available at https://github.com/mhoubraken/ISMAGS.

The experiments were performed on a single core of an Intel Core 2 Duo P8400 processor clocked at 2.26 GHz with 4 GB of RAM under a 64-bit Windows installation. To remove the influence of memory operations, the reported times exclude the reading of the networks and writing to memory of the subgraph instances found. The times reported in the results thus only pertain to the time needed to look for all possible matches in the search space and, if applicable, the time needed to analyse the subgraph for symmetries. Most of the results were averaged over 1000 runs. However, some test instances were limited to fewer runs as the long calculation times were prohibitive for more elaborate testing. The number of runs used for averaging is shown along with the results. When fewer runs were used, this is denoted with an asterisk or circle, depending on the number of runs.

### Network data

The input networks for the experiments are similar to the networks from [14]. They are denoted as biological, Slashdot and SNAP (based on the source of the data) and their properties can be found in Table 5. Note that while only results are shown for these 3 types of networks, ISMAGS can be used for subgraph matching in any graph with multiple edge types.

**Table 5:** Network properties.

Network	#Nodes	#Edges
PGS (reduced)	1255	6454
P	887	1844
G	469	4051
S	404	659
XYZ/ABZ	15078	79794
X/A	4847	36391
Y/B	9602	40630
Z	5208	3132
Slashdot	79120	469768
E	37412	118755
F	69998	351013
Wiki-Vote	7115	100762
p2p-Gnutella08	6301	20777
p2p-Gnutella30	36682	88328
CA-CondMat	23133	93439
CA-HepTh	9877	25973

For each network, the number of nodes and edges is given. If multiple edge types are present in the network, separate counts are given for each edge type, denoting the number of edges of the specific type as well the number of nodes having an edge of that type. The XYZ-network and the ABZ-network have the same node and edge count as the A- and B-edges are the directed versions of the X- and Y-edges. Similarly, the reduced PGS-network has the same node and edge count as the PGS-network.

The biological networks comprise two networks with multiple edge types. The first network pertains to physical (P, undirected), genetic (G, undirected) and signalling (S, directed) interactions between kinases and phosphatases in yeast [19], [20]. The second network consists of protein-protein interaction in yeast (X, undirected, obtained from the BioGRID [21] database), protein-protein interactions in humans (Y, undirected, obtained from the BioGRID and STRING [22] databases), and orthology relations between human and yeast proteins (Z, bipartite, from the InParanoid database [23]). Additionally, both networks were modified to obtain additional test networks. The reduced PGS-network is constructed by interpreting all edges in the PGS-network to be undirected and of the same edge type. The ABZ-network is derived from the input files of the XYZ-network. The X- and Y-edges, specified as “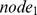 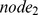
” on individual lines in their respective edge file, are interpreted as directed A- and B-edges going from 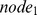 to 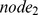.

The Slashdot network [24] represents “friend” and “foe” relations between users of the technology-centred Slashdot community. A group of friends in which everyone is a friend of each other can be represented as an F-clique subgraph while two friends with a mutual enemy can be represented by a “FEE” subgraph. Note that the edges are assumed to be undirected. Finding subgraphs in this social network is an example of how ISMAGS can be used to mine social data.

The third set of networks consists of some of the networks available in the SNAP database (found at http://snap.stanford.edu/data/). The Wiki-Vote network represents votes cast by users during Wikipedia admin elections, the p2p-Gnutella08 and p2p-Gnutella30 are 2 snapshot of the Gnutella peer-to-peer network and the CA-CondMat and CA-HepTh networks are collaborations networks based on co-authorship of papers published in the arXiv repository in the Condense Matter and the High Energy Physics - Theory category, respectively. For these networks, the 3- and 4-node cliques are searched as well as the instances of the subgraphs “XxXXXX” (tetrahedron) and “xXxXxx” (G4). Note that, during the search for the instances of the cliques, the edges in the networks are considered to be undirected while they are considered to be directed during the search for the instances of the tetrahedron and the G4 graphs. This difference in interpretation allows to show the performance of the symmetry handling of the algorithms on both directed and undirected networks. The tetrahedron subgraph was selected to be searched for as it contains a relatively simple symmetric structure that was not taken into account in ISMA. The G4 graph was selected to show the effects of the optimisation of the set operations in ISMAGS (compared to ISMA) as it does not have any symmetric properties.

Note that the number of nodes and edges reported in Table 5 can differ from the counts of the original data source. This is a result of network preprocessing. Aside from removing unconnected nodes and (anti-)parallel edges, the preprocessing also ensured that only 1 edge is present between any pair of nodes. While ISMAGS correctly deals with these issues, not all of the benchmark implementations support them. The preprocessed networks are included in the source code of ISMAGS (available at https://github.com/mhoubraken/ISMAGS).

### ISMA versus ISMAGS

To show the advantages of incorporating the symmetric information in the search, Table 6 compares the performance of ISMA to ISMAGS on the biological networks. The search space reduction factor (SPRF) varies depending on the subgraph.

**Table 6:** Comparison between ISMA and ISMAGS on the biological networks.

		Search space (#nodes)			Calculation time (ms)		
	#instances	ISMA	ISMAGS	SPRF	ISMA	ISMAGS	SF
PGS-network							
GGG	9008	4520	4520	1.00	25.91	5.80	4.46
SSS	78	763	761	1.00	1.61	0.24	6.65
SsS	0	190	90	2.11	0.62	0.06	10.24
GPS	47	462	454	1.02	0.99	0.28	3.56
SSG	103	763	761	1.00	1.76	0.44	3.97
SsG	25	190	131	1.45	0.49	0.13	3.79
GGS	294	462	454	1.02	1.29	0.48	2.69
GGP	418	8571	8571	1.00	14.96	3.83	3.90
ssG	112	372	419	0.89	0.91	0.25	3.69
PGSPGS	0	391	232	1.69	0.97	0.14	7.02
P0P	24452	4575	4575	1.00	19.59	2.87	6.82
P0P00P	221290	57167	53479	1.07	192.67	30.67	6.28
P0P00P000P	2570154	551837	496059	1.11	2142.74	302.55	7.08
Petersen	9430	1131600	616418	1.84	330882*	733.25	451
Reduced PGS-network							
3-clique	10614	7709	7709	1.00	39.50	8.81	4.48
4-clique	11150	18323	18323	1.00	118.69	27.60	4.30
5-clique	7669	29473	29473	1.00	225.49	48.36	4.66
6-clique	3616	37142	37142	1.00	320.71	64.90	4.94
7-clique	1158	40758	40758	1.00	379.00	76.63	4.95
8-clique	226	41916	41916	1.00	412.69	84.97	4.86
9-clique	24	42142	42142	1.00	431.79	92.21	4.68
10-clique	1	42166	42166	1.00	451.08	99.08	4.55
XYZ-network							
XZ00ZY	2554	23164	19095	1.21	36.66	9.58	3.83
XXXZ000Z0Y00ZYY	4727	73647	39572	1.86	152.77	27.54	5.55
ABZ-Network							
AZ00ZB	1337	11588	10859	1.07	20.46	6.02	3.40
AAAZ000Z0B00ZBB	837	15186	14190	1.07	36.52	9.73	3.75

The reported #instances is the number of subgraph instances as exported by ISMAGS. The search space size is the #nodes visited during the search process. SPRF is the search space reduction factor and is calculated as the ratio of the search space size for ISMA to the search space size for ISMAGS. The speed-up factor is defined analogously for the calculation time. All reported timings are averaged over 1000 runs unless denoted by an asterisk *, in which case only one test was performed as the long computation times were prohibitive for more elaborate testing.

For the subgraphs with 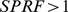, the reduction can be attributed to various factors. For the subgraphs with limited symmetry (“SsS”, “SsG”, “PGSPGS”), the reduction is due to a quicker termination of uninteresting paths. When the mapping of a node would result in empty candidate lists for an unmapped node, this is detected in ISMAGS before the node is mapped and can quickly be terminated. For the subgraphs with large symmetric structures (Petersen graph of Fig. 1, XYZ subgraphs), the reduction comes from the symmetry-handling which was not fully exploited in ISMA, showing the benefits of the improved symmetry-breaking approach.

For the “ssG” subgraph, a small increase in search space is present. This is due to the omission of list membership tests of the large initial lists of candidates. While omitting these tests allows to map candidates faster, it increases the search space slightly as some graph nodes get examined while they do not have all required edges. However, the speed gain of omitting these tests still outweighs the slight increase.

The SPRFs around 1 indicate that ISMA and ISMAGS follow the same path through the search space. This is primarily the case when the symmetry in the subgraphs is incorporated in ISMA (e.g. “GGG”, “SSS”, cliques). Interestingly, ISMAGS still reduces query times for these subgraphs due to the list-based implementation of its symmetry breaking. When calculating candidate sets for the subgraph nodes involved in the symmetric structures, ISMA removes nodes from constraints sets before calculating the intersections of its sets. This ensures that no nodes get mapped in symmetric configurations. However, to calculate the intersection, ISMA still needs to intersect the different sets. ISMAGS uses the ID-based constraints derived during symmetry analysis to avoid most work during list intersection. Using the constructed partial mapping and the constraints, ISMA can quickly find the interesting range of nodes in the to-be-intersected lists and ignore the remainder. This reduces the time needed for candidate set generation and improves execution time results. Additionally, as explained above, ISMAGS omits the large initial lists of candidate graph nodes when calculating candidate set lists once the initial node is determined. This reduces calculation time as less list membership needs to be checked.

The speed-up factor in Table 6 shows the ratio of the calculation time of ISMA to the calculation time of ISMAGS. For most investigated subgraphs, the speed-up factor is larger than 3, indicating that ISMAGS only needs a third of the calculation time of ISMA. While ISMAGS gives a speed-up for the subgraphs previously published [14] due to the more optimised list-based implementation, it was designed to handle more complex symmetries in the subgraphs. This can be seen in the results of the Petersen graph and the line graphs. The Petersen graph [25] is inherently very symmetric as one instance can be permuted in 120 other instances. The line graphs (“P0P”, “P0P00P” and “P0P00P000P”) consist of 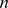 nodes, connected by 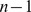 edges, with node 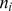 connected to 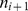 for 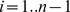. As such, the line graphs correspond to chains of nodes. The symmetry in these graphs is limited to reversing the chains, as every subgraph instances can be read left-to-right and right-to-left. While this is a simple symmetry, it involves multiple nodes being remapped, which was not fully incorporated in ISMA.

An analogous analysis was done on the Slashdot and SNAP networks and can be found in Table 7. The test instances with 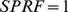 (cliques) have similar search spaces between the 2 algorithms. For the G4 graph, the 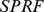 slightly increases, indicating that ISMAGS mostly traverses the same search space as ISMA (as no symmetry could be exploited), with the exception of a few optimised list selections. The results for the tetrahedron show a 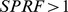, indicating that the symmetric structure in the tetrahedron is taken into account by ISMAGS but not by ISMA. The speed-up factors are slightly lower for the Slashdot test instances than for the biological and SNAP networks. This is mostly due to the fact that ISMA already incorporates most of the symmetric information in the subgraphs and thus leaves less room for improvement for ISMAGS. However, ISMAGS still speeds up the searches. The speed-up factors for the 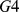 graph are also slightly lower than for the other graphs as no symmetry could be used to speed up the search.

**Table 7:** Comparison between ISMA and ISMAGS on the Slashdot and SNAP networks.

		Search space (#nodes)			Calculation time (ms)		
	#instances	ISMA	ISMAGS	SPRF	ISMA	ISMAGS	SF
Slashdot							
3-clique E	12176	156167	156167	1.00	844.37	283.44	2.98
4-clique E	223	168343	168343	1.00	1075.89	364.76	2.95
5-clique E	3	168566	168566	1.00	1253.54	392.47	3.19
3-clique F	392556	421011	421011	1.00	4145.04	1600.07	2.59
4-clique F	1779701	813567	813567	1.00	14137.33	4727.03	2.99
FEF	67594	148084	139542	1.06	1018.42	890.77	1.14
FEE	75100	178997	173435	1.03	1145.29	618.22	1.85
FE00EF	755899	7544696	1577058	4.78	35769.79	5388.92	6.64
Wiki-Vote							
3-clique	608389	107877	107877	1.00	1794.22	410.19	4.37
4-clique	2077903	716266	716266	1.00	14137.27	5156.31	2.74
tetrahedron	84787	115205	49836	2.31	1025.65	320.72	3.20
G4	62406	80584	76364	1.06	580.07	448.54	1.29
p2p-Gnutella08							
3-clique	2383	27078	27078	1.00	84.96	21.23	4.00
4-clique	175	29461	29461	1.00	107.87	28.69	3.76
tetrahedron	2	9565	6043	1.58	21.22	4.92	4.32
G4	6	9546	9472	1.01	20.03	7.97	2.51
p2p-Gnutella30							
3-clique	1590	125010	125010	1.00	405.26	113.69	3.56
4-clique	13	126600	126600	1.00	464.50	139.06	3.34
tetrahedron	2	41147	27365	1.50	101.60	28.03	3.62
G4	0	41149	40910	1.01	99.60	38.12	2.61
CA-CondMat							
3-clique	173361	116572	116572	1.00	548.19	128.96	4.25
4-clique	294008	289933	289933	1.00	1518.38	357.29	4.25
tetrahedron	0	67688	39758	1.70	163.95	35.08	4.67
G4	0	62974	62612	1.01	149.69	54.06	2.77
CA-HepTh							
3-clique	28339	35850	35848	1.00	118.27	30.45	3.88
4-clique	65592	64187	64187	1.00	290.38	64.56	4.50
tetrahedron	0	18258	11434	1.60	37.38	9.16	4.08
G4	0	17853	17068	1.05	35.82	11.54	3.10

The reported parameters are analogously defined as in Table 6. Note that for the clique subgraphs, the SNAP networks are assumed to be undirected, while for the tetrahedron, they are assumed to be directed.

### Full comparison

In addition to ISMA, the ISMAGS algorithm was compared to 3 similar algorithms, viz the VF2, the GK and the G-Trie algorithm. Tables 8 and 9 show the timing results of the VF2 algorithm, the subgraph enumeration algorithm from [7] and the G-Trie algorithm for some of the subgraphs from the previous section along with the results of ISMAGS. Note that not all test instances from the previous table are repeated as the reference algorithms implementations, in contrast to ISMA and ISMAGS, were not designed to support multiple edges (of different types) between a pair of nodes.

**Table 8:** Comparison between ISMAGS, VF2, GK and G-Trie on the biological networks.

		Calculation time (ms)			
	#instances	VF2	GK	G-Trie	ISMAGS
#runs		1000	1000	1000	1000
PGS-network					
GGG	9008	885.56	319.27	1.03	5.80
SSS	78	24.12	22.90	0.25	0.24
SsS	0	22.50	17.40	0.22	0.06
P0P	24452	268.76	103.81	-	2.87
P0P00P	221290	4252.12	585.77	-	30.67
P0P00P000P	2570154	35303*	5705.64	-	302.55
Petersen	9430	6854418*	53608*	-	733.25
Reduced PGS-network					
3-clique	10614	1170*	511.33	1.51	8.81
4-clique	11150	7300*	1177.12	3.60	27.60
5-clique	7669	32527*	2160.31	5.55	48.36
6-clique	3616	115409*	2968.93	7.44	64.90
7-clique	1158	329521*	3513.20	14.41	76.63
8-clique	226	754671*	3700.21	71.60	84.97
9-clique	24	1337881*	3813.57	671.08	92.21
10-clique	1	1848315*	4181.80	-	99.08

The top row denotes, for each algorithm, the number of runs averaged to obtain the reported timing results. However, for results denoted with an asterisk, only 1 run was performed. For the G-Trie algorithm, some results are missing as the size of the subgraph ( = 10 nodes) was not supported by the reference implementation. The results for the “P0P”, “P0P00P” and “P0P00P000P” are also omitted as the algorithm did not support “don't care” links.

**Table 9:** Comparison between ISMAGS, VF2, GK and G-Trie on the SNAP networks.

		Calculation time (ms)			
	#instances	VF2	GK	G-Trie	ISMAGS
#runs		100	100	1000	1000
Wiki-Vote					
3-clique	608389	187191.52	27940.99	90.28	410.19
4-clique	2077903	3410302°	189357.30	613.08	5156.31
tetrahedron	84787	15260.17	106367.64	443.71	320.72
G4	62406	8168.52	128836.22	1006.36	448.54
p2p-Gnutella08					
3-clique	2383	816.04	1163.03	6.66	21.23
4-clique	175	1659.69	1359.35	6.81	28.69
tetrahedron	2	114.66	1151.98	6.18	4.92
G4	6	108.73	1766.08	12.32	7.97
p2p-Gnutella30					
3-clique	1590	6259.23	5681.83	43.21	113.69
4-clique	13	5867.19	5527.54	43.07	139.06
tetrahedron	2	1991.82	5793.46	34.34	28.03
G4	0	1964.98	6671.50	72.07	38.12
CA-CondMat					
3-clique	173361	37742.10	7196.78	41.73	128.96
4-clique	294008	232134.17	15558.19	68.48	357.29
tetrahedron	0	6547.67	11779.51	40.48	35.08
G4	0	4848.04	13660.84	121.68	54.06
CA-HepTh					
3-clique	28339	4441.81	1416.95	7.64	30.45
4-clique	65592	21374.00	2361.03	11.19	64.56
tetrahedron	0	539.45	1790.64	6.65	9.16
G4	0	392.03	2449.13	19.03	11.54

Similar to Table 8, the top row denotes, for each algorithm, the number of runs averaged to obtain the reported timing results. However, the result denoted with a circle was averaged over 10 runs.

Table 8 shows the results for the test instances on the biological networks. Compared to the VF2 algorithm, the GK algorithm reduces query times by exploiting the symmetry in the cliques. As the cliques have more nodes, the symmetry breaking increasingly prunes the search space (e.g. for the 10-clique, only 1 match out of 10! permutations is retained). ISMAGS however further reduces computation times by the optimised matching processes and symmetry breaking described in the previous sections. For most instances, ISMAGS reduces query times by 1–2 orders of magnitude compared to VF2 and the GK algorithm.

Of the reference algorithms, the G-Trie algorithm was the only one able to match the performance of ISMAGS. While G-Trie performs very well for the clique graphs, it was not able to process the large subgraphs as ISMAGS did. Results are also missing for the line graphs (“P0P”, “P0P00P” and “P0P00P000P”) and the Petersen graph as G-Trie expects all edges to be defined as present or absent while in ISMAGS undefined edges are treated as “don't care” ( = can be present or absent).

The results for the instances on the SNAP networks are shown in Table 9. Compared to VF2 and GK, ISMAGS again significantly reduces query times by 1–2 orders of magnitude. This allows to process larger networks in reasonable time frames and opens up possibilities for research. Interestingly, the GK results for the tetrahedron instance are worse than the VF2 algorithm. This can be explained by the fact that the GK algorithm focuses on reducing the search space while VF2 focuses on search space traversal. GK reduces the search space by a factor of 3 (by exploiting the symmetry of the tetrahedron) but the VF2 more efficiently processes its (larger) search space. For larger subgraphs, GK becomes better as the symmetry breaking can prune more search space.

While the G-Trie algorithm performs well for clique graphs, ISMAGS performs better for the tetrahedron and the G4 graph. This is due to the dynamic node order in the search space traversal. While this optimisation is one of the primary features of ISMA and ISMAGS, the order in which the nodes are mapped in G-Trie is fixed to accommodate matching multiple subggraphs at the same time. This gives ISMAGS the advantage when matching single subgraphs with varying neighbour list sizes.

ISMAGS was implemented in Java, a high level programming language with the advantages of dynamic memory management, fast implementation, easily accessible (i.e. readable) code and portability. However, additional speed-up could be gained by implementing ISMAGS in C(++), as is the case for G-Trie, but at the cost of losing the flexibility and portability.

## Conclusions

In this paper, we present the Index-Based Subgraph Matching Algorithm with General Symmetries (ISMAGS), an improved version of the Index-Based Subgraph Matching Algorithm (ISMA). The improved version takes into account all symmetric structures in a subgraph. Whereas ISMA minimises the search space exploration by optimising the order in which the nodes of the query subgraph are investigated, it only takes into account the basic symmetries (e.g. single node symmetry and rotation). ISMAGS removes this restriction by introducing symmetry-breaking constraints in the search tree traversal using a customised symmetry analysis. This analysis yields symmetry-breaking constraints which were incorporated in a list-based implementation of the algorithm. Experiments show that the optimised implementation of list operations and the symmetry-breaking constraints significantly reduce calculation times. On average, a speed-up factor (compared to ISMA) of 3 to 4 was present for the subgraphs in the experiments. However, depending on the degree and complexity of symmetry in the subgraph, the speed-up factor varied between 1.14 (for simple symmetric structures already incorporated in ISMA) to 451 (for complex symmetries). Compared to the VF2 and GK algorithms available in literature, ISMAGS also reduces query times by 1 to 2 orders of magnitude. ISMAGS' performance is more closely matched to the G-Trie algorithm but the latter does not perform well on larger subgraphs and cannot take advantage of ordering nodes dynamically. While ISMAGS was initially developed to speed up finding subgraph instances in biological networks with multiple edge types, the algorithm can also be used in non-biological networks like social networks to speed up network analysis (e.g. mining for social structures).
